# Staged Management of an Open Bimalleolar Ankle Fracture Combined With Calcaneum and Lisfranc Injuries: A Rare Presentation

**DOI:** 10.7759/cureus.116140

**Published:** 2026-09-12

**Authors:** Narendra Kumar, Mohit Patralekh, Vipul Pathak, Bharat Sharma, Hirdyanshu Pundhir

**Affiliations:** 1 Central Institute of Orthopaedics, Vardhman Mahavir Medical College & Safdarjung Hospital, New Delhi, IND

**Keywords:** calcaneal fracture, complex foot and ankle trauma, damage control orthopedics, gustilo-anderson type iiib, lisfranc fracture-dislocation, negative pressure wound therapy (npwt), open bimalleolar ankle fracture, spanning external fixation, split-thickness skin graft (stsg), staged management

## Abstract

The simultaneous occurrence of an open bimalleolar ankle fracture with ipsilateral calcaneal fracture and Lisfranc fracture-dislocation within a single extremity represents an extraordinarily rare constellation of injuries resulting from high-energy trauma. When associated with a Gustilo-Anderson Type IIIB soft-tissue defect, traditional open reduction and internal fixation (ORIF) carries an increased risk of deep hardware infection, wound breakdown, and osteomyelitis. The extensive soft-tissue injury further complicates management and may preclude immediate internal fixation.

This report demonstrates the successful implementation of a staged management strategy that prioritized soft-tissue preservation over immediate rigid osteosynthesis. The patient achieved a functional, plantigrade limb and was able to perform activities of daily living and return to vocational activity, with an AOFAS score of 78 at six months of follow-up.

## Introduction

High-energy trauma to the lower extremity generally results in complex fracture patterns. However, the simultaneous occurrence of a bimalleolar ankle fracture, calcaneal fracture, and Lisfranc injury in the same limb is very unusual. This triad indicates a catastrophic transfer of kinetic energy, speculated to result from a combination of axial loading, hyperplantarflexion, and rotational crush forces.

When accompanied by an open soft-tissue injury, the orthopedic surgeon faces a dual challenge: achieving functional biomechanical alignment while preventing deep infection and soft-tissue necrosis. Traditional open reduction and internal fixation (ORIF) is often contraindicated in such circumstances because of the compromised soft-tissue envelope. This article describes the successful staged management of this unique complex trauma using an external fixator, percutaneous K-wires, and eventual soft-tissue coverage with a split-thickness skin graft (STSG).

## Case presentation

We received the case of a 40-year-old man who sustained a severe open bimalleolar ankle fracture, an ipsilateral calcaneal fracture, and a Lisfranc fracture-dislocation following a high-velocity motor vehicle collision in which a truck struck a helmeted motorcycle rider. On local examination of the left lower limb, the foot and ankle were grossly deformed, with a degloving wound measuring 15 × 8 cm over the dorsum of the foot and extending to the lateral aspect of the ankle. There was also avulsion of the great toenail with exposed bone. The injury was classified as a Gustilo-Anderson type IIIB injury (Figure 1).

**Figure 1:**
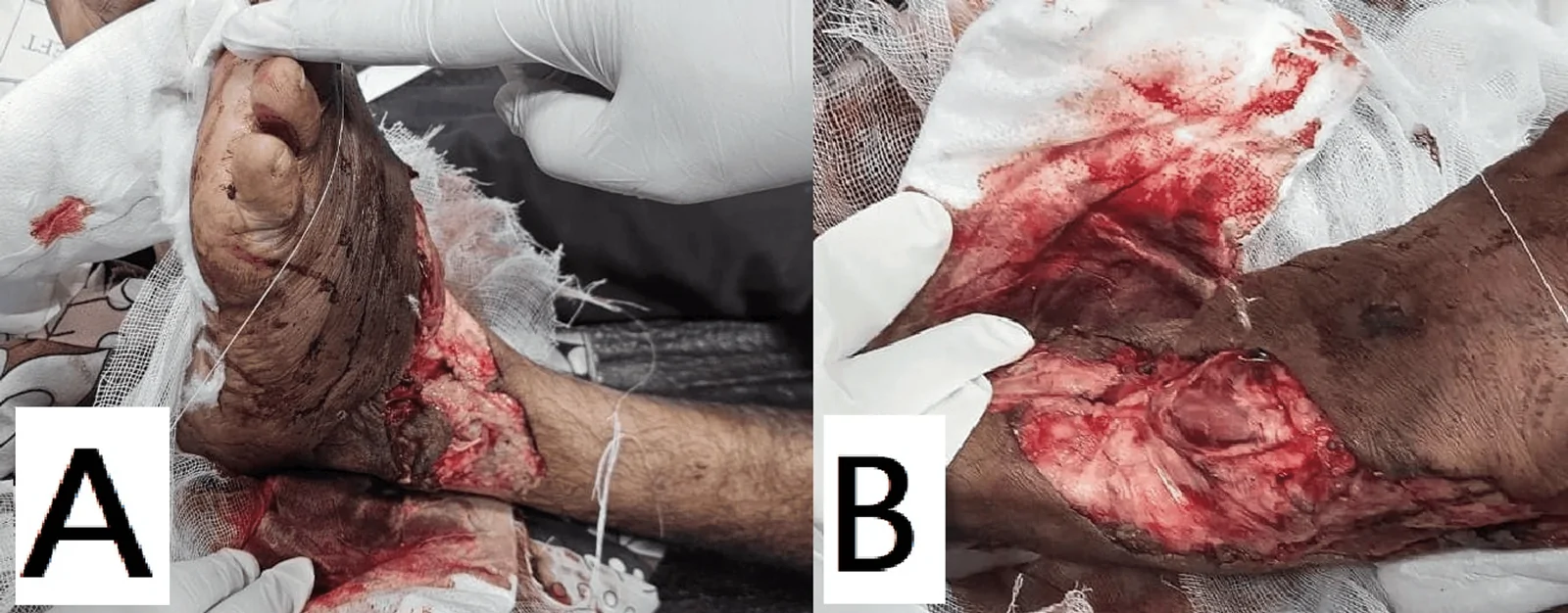
Clinical photographs at initial presentation of severe, high-energy open lower-extremity trauma (A) Lateral aspect of left ankle and foot. (B) Medial aspect of the left ankle and foot before surgical debridement and skeletal stabilization.

The wound exhibited significant soft-tissue stripping. The dorsalis pedis and posterior tibial pulses were not palpable; however, capillary refill and pinprick sensation were intact. Plain radiographs and subsequent arterial Doppler ultrasonography of the foot and ankle were obtained. Imaging demonstrated a displaced bimalleolar fracture of the ankle, a minimally displaced extra-articular fracture of the calcaneus with an intact lateral facet in the hindfoot, a divergent Lisfranc fracture-dislocation of the midfoot, and a transverse fracture of the proximal phalanx of the great toe in the forefoot (Figure 2).

**Figure 2:**
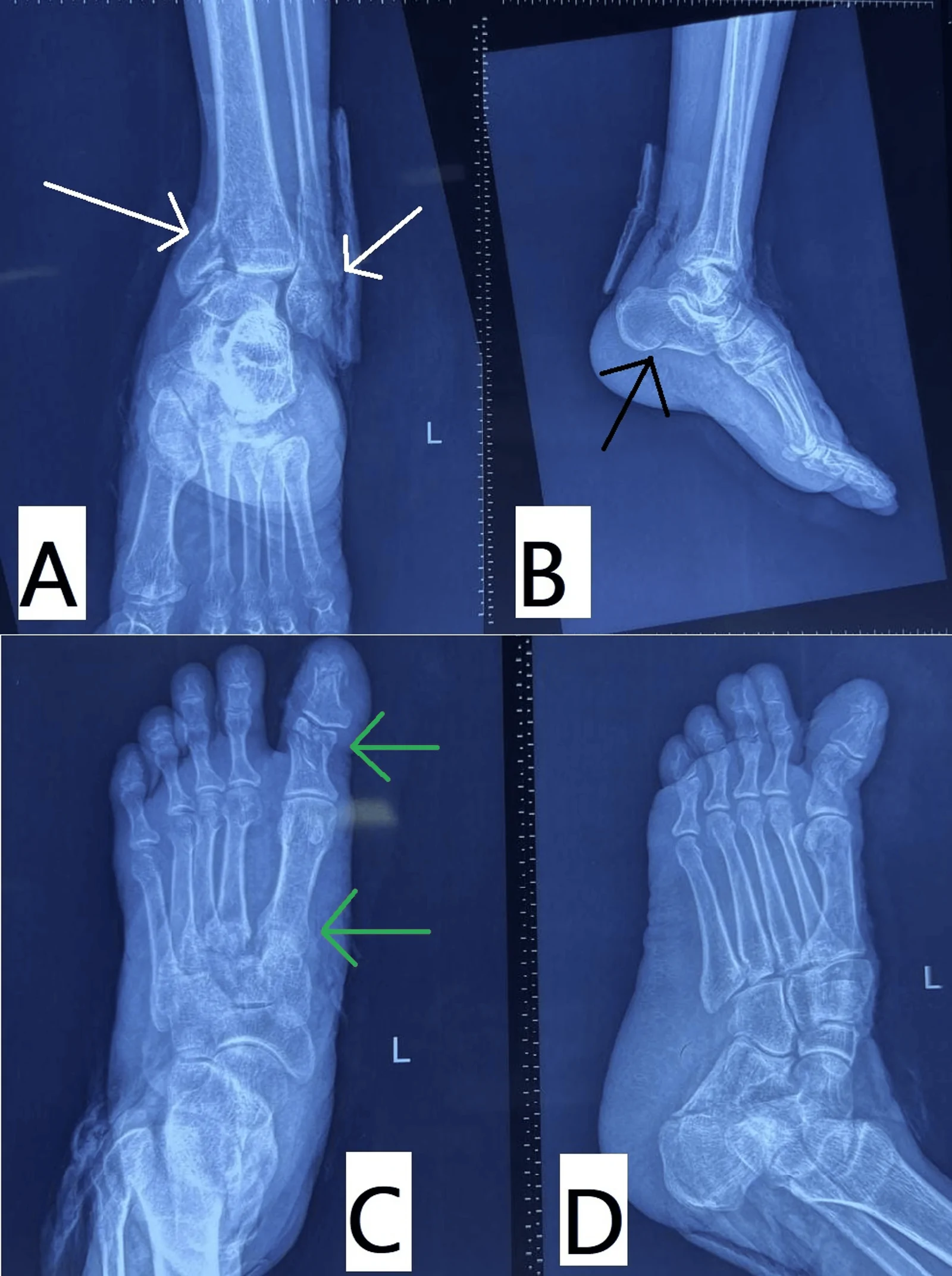
Radiographs at presentation (A) Anteroposterior (AP) view of the ankle. (B) Lateral view of the ankle. (C) AP view of the foot. (D) Oblique view of the foot showing a displaced bimalleolar fracture of the ankle joint (white arrows; A), a minimally displaced extra-articular fracture of the calcaneus (black arrow; B), a divergent Lisfranc fracture-dislocation with lateral displacement of the lesser metatarsals and widening of the first intermetatarsal space, and a transverse fracture of the proximal phalanx of the great toe (green arrows; C).

Arterial Doppler ultrasonography revealed multiphasic flow in the anterior tibial artery (ATA), posterior tibial artery (PTA), and dorsalis pedis artery (DPA) of the left lower extremity.

Given the severe soft-tissue compromise, a damage-control orthopedic approach was elected. The patient underwent initial debridement and skeletal stabilization during emergency surgery. Radical irrigation and debridement of the open wound were performed to remove all necrotic and devitalized tissue. To minimize further insult to the soft-tissue envelope, extensive surgical approaches were avoided. The Lisfranc joint complex was reduced through the open wound and stabilized using multiple percutaneous K-wires. Bimalleolar instability and overall foot and ankle instability were managed with percutaneous K-wires to maintain the reduction, followed by application of an ankle-spanning delta external fixator with pins placed in the first and fifth metatarsals. The external fixator was applied across the ankle joint, extending from the tibia to the forefoot, and maintained the foot in a neutral position. The calcaneal fracture was minimally displaced and did not warrant stabilization with K-wires because of the presence of open wounds and the preservation of articular congruity with an intact lateral facet (Figure 3).

**Figure 3:**
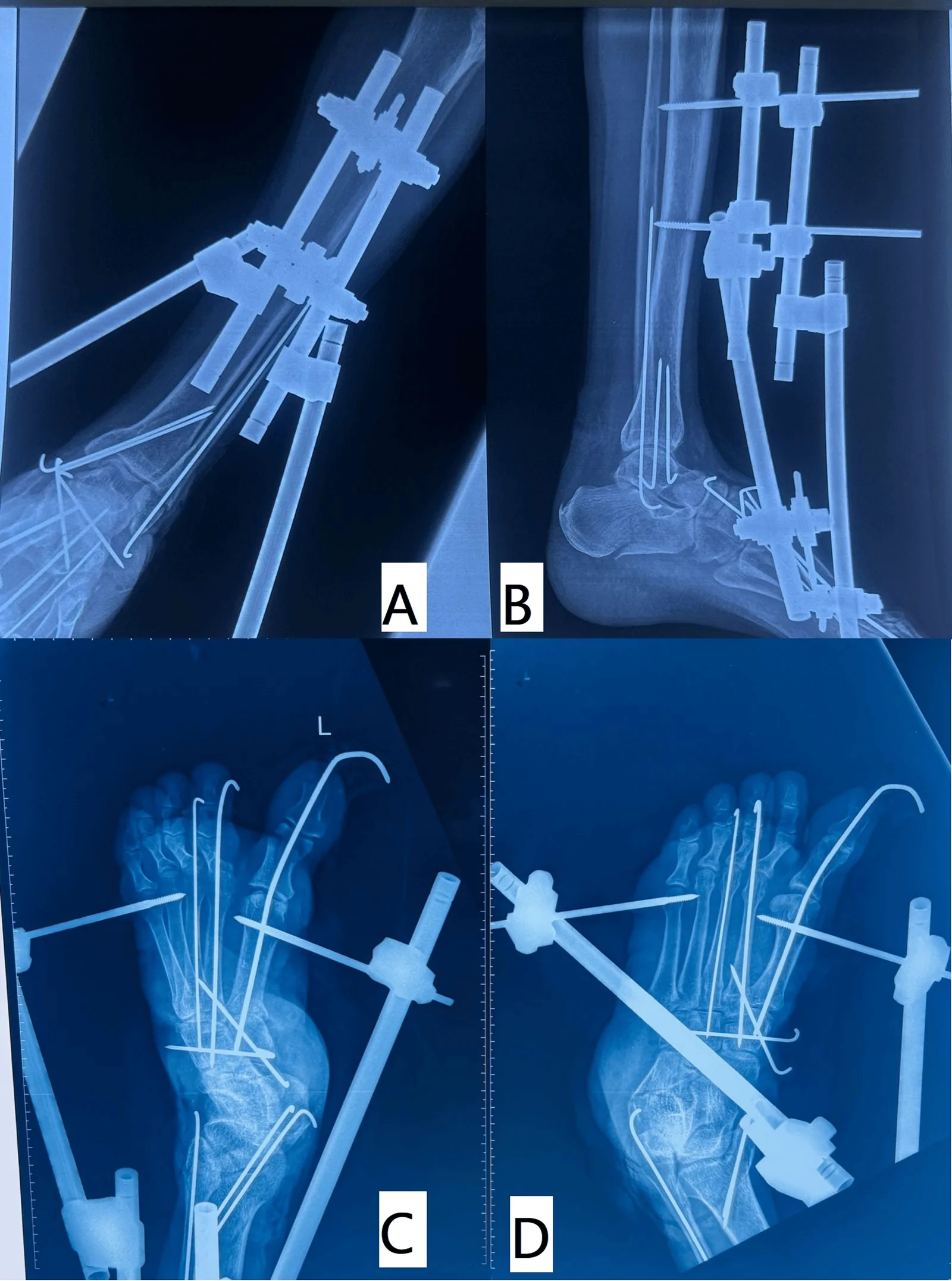
Radiograph at postoperative day 1 (A) AP view of ankle (B) Lateral view of ankle (C) AP view of foot (D) Oblique view of foot showing successful application of stabilization construct using percutaneous Kirschner wires (K-wires) and an ankle-spanning external fixator.

Distal pulses were palpable following emergency surgery.

Soft-tissue reconstruction was subsequently performed four weeks later. In the interim, the open wound was managed with regular dressing changes, followed by application of negative pressure wound therapy (NPWT). By four weeks, a healthy, robust granulation bed had developed over the defect. The patient was then taken back to the operating room for STSG, with the graft harvested from the contralateral thigh (Figure 4).

**Figure 4:**
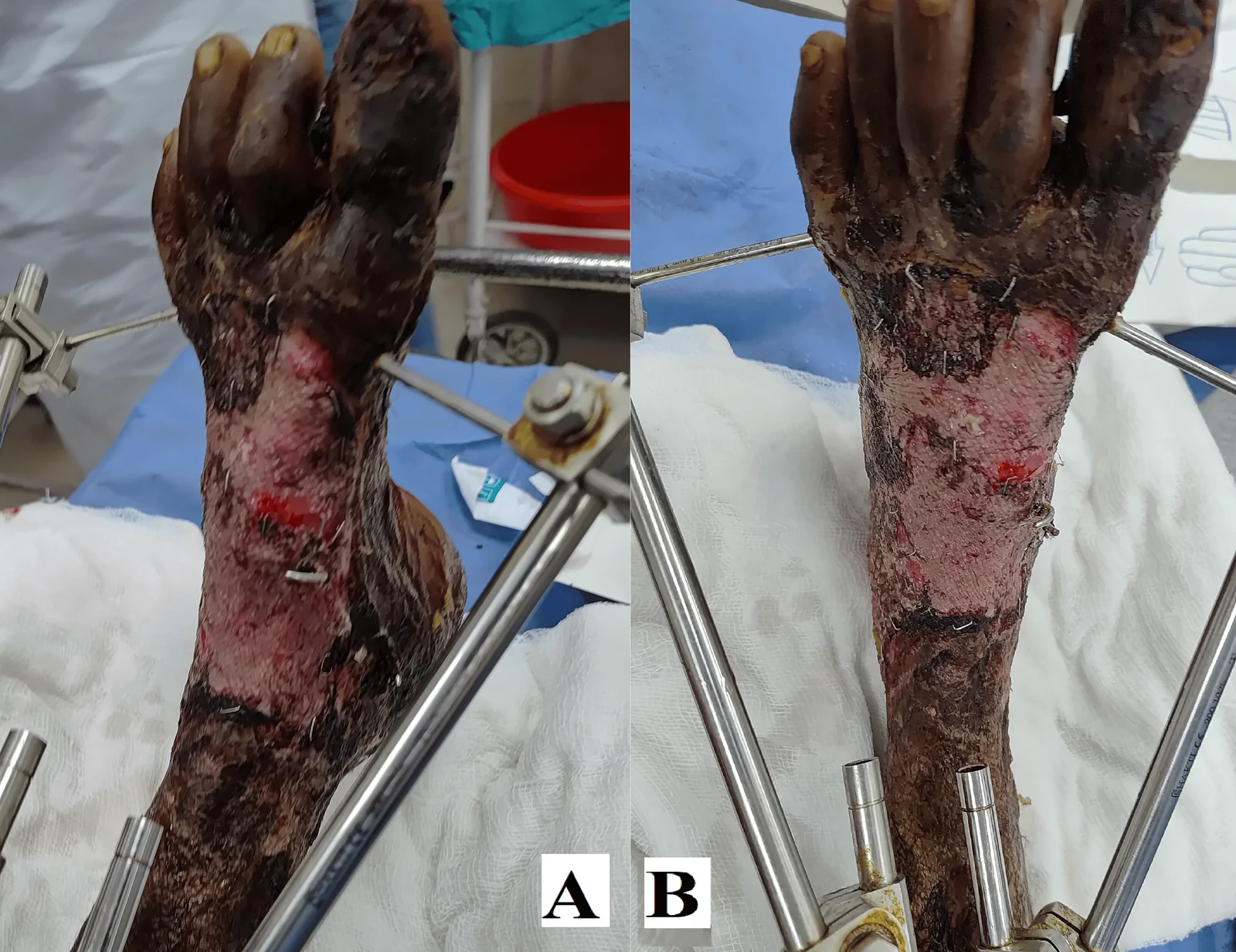
Clinical photographs after split-thickness skin graft application (A) Medial aspect and (B) dorsal aspect of the left foot and ankle showing successful application of a split-thickness skin graft (STSG) over the previously degloved wound on the dorsum of the foot, with the ankle-spanning external fixator and K-wires in place.

The graft demonstrated successful take, providing durable coverage over the previously exposed ankle joint.

For continued stabilization and subsequent hardware removal, the external fixator and K-wires were maintained to allow simultaneous bony consolidation. Serial radiographs demonstrated progressive callus formation with maintained articular alignment. At six weeks postoperatively, clinical and radiological evidence of union of the ankle and calcaneal fractures, along with adequate integration of the STSG, was considered sufficient, and the spanning external fixator was removed (Figure 5).

**Figure 5:**
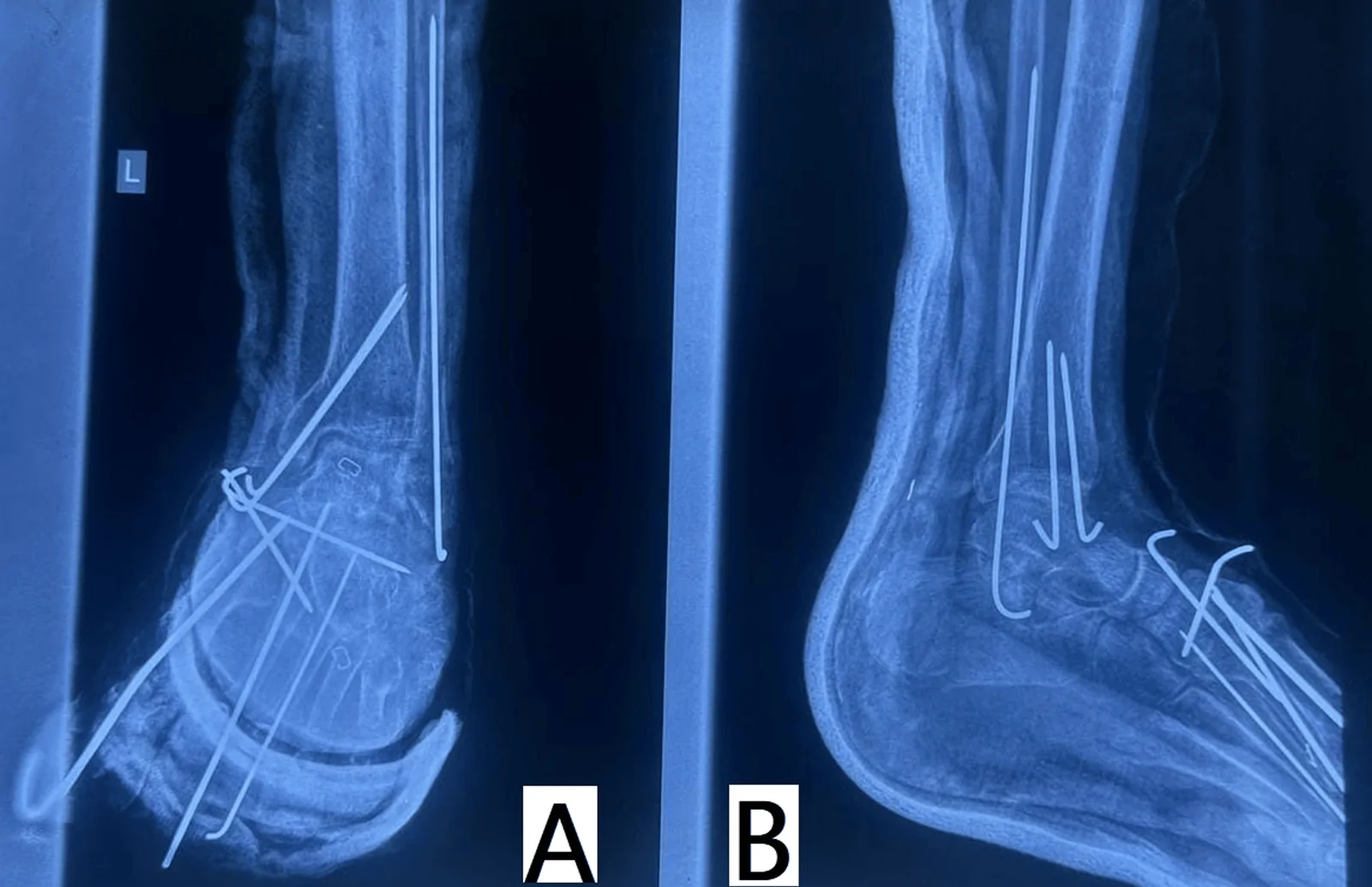
Radiographs at six weeks postoperatively after removal of the external fixator (A) Anteroposterior (AP) view and (B) lateral view of the ankle demonstrating progressive healing of the fractures.

At eight weeks postoperatively, the Lisfranc K-wires were removed in the operating room to allow motion of the midfoot and forefoot (Figure 6).

**Figure 6:**
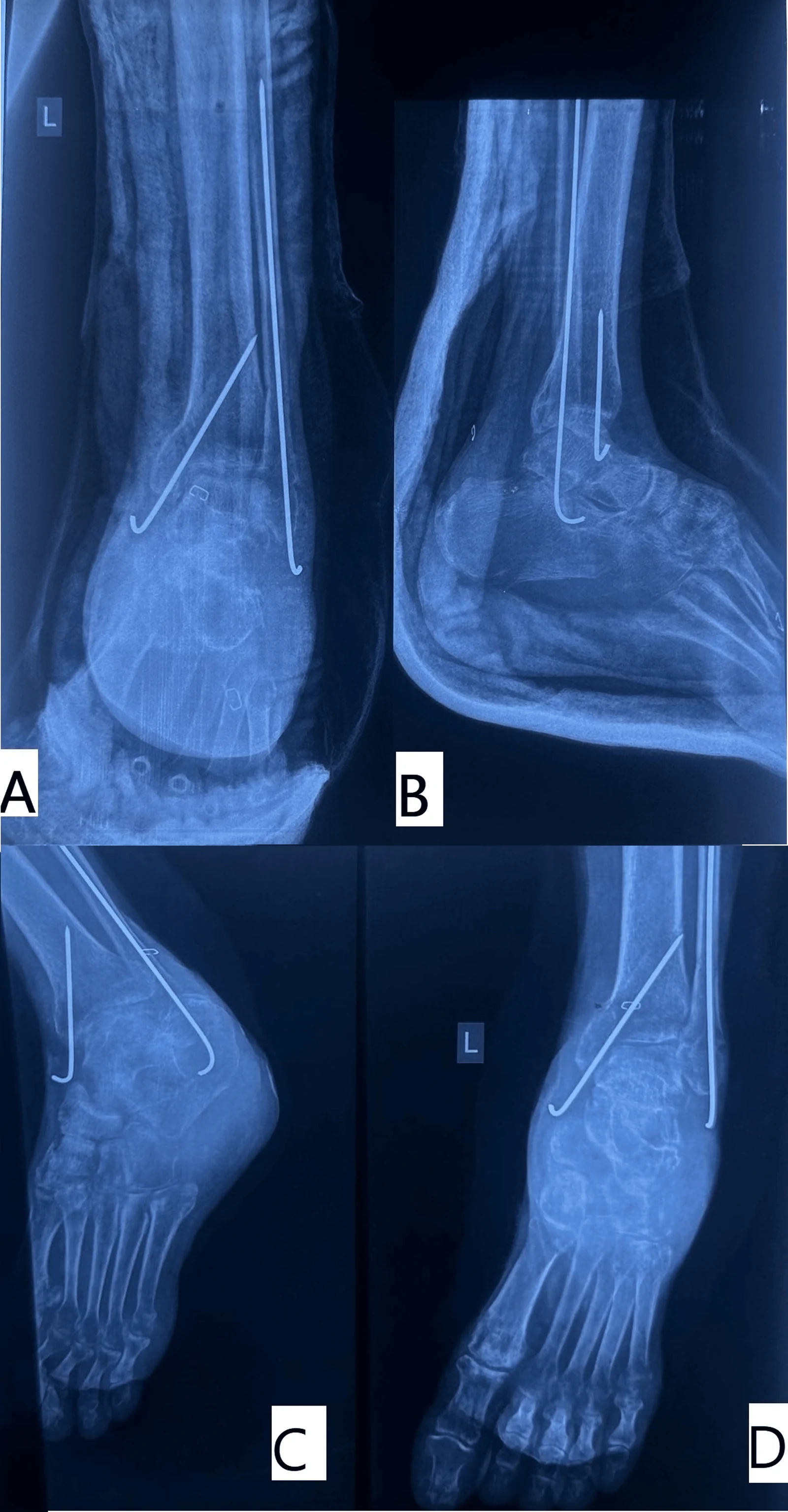
Radiographs at 8 weeks postoperatively (A) Anteroposterior (AP) view of the ankle. (B) Lateral view of the ankle. (C) Oblique view of the foot. (D) AP view of the foot demonstrating fracture healing following removal of the K-wires from the foot.

At 10 weeks postoperatively, following satisfactory union of both malleoli, the malleolar K-wires were removed (Figure 7).

**Figure 7:**
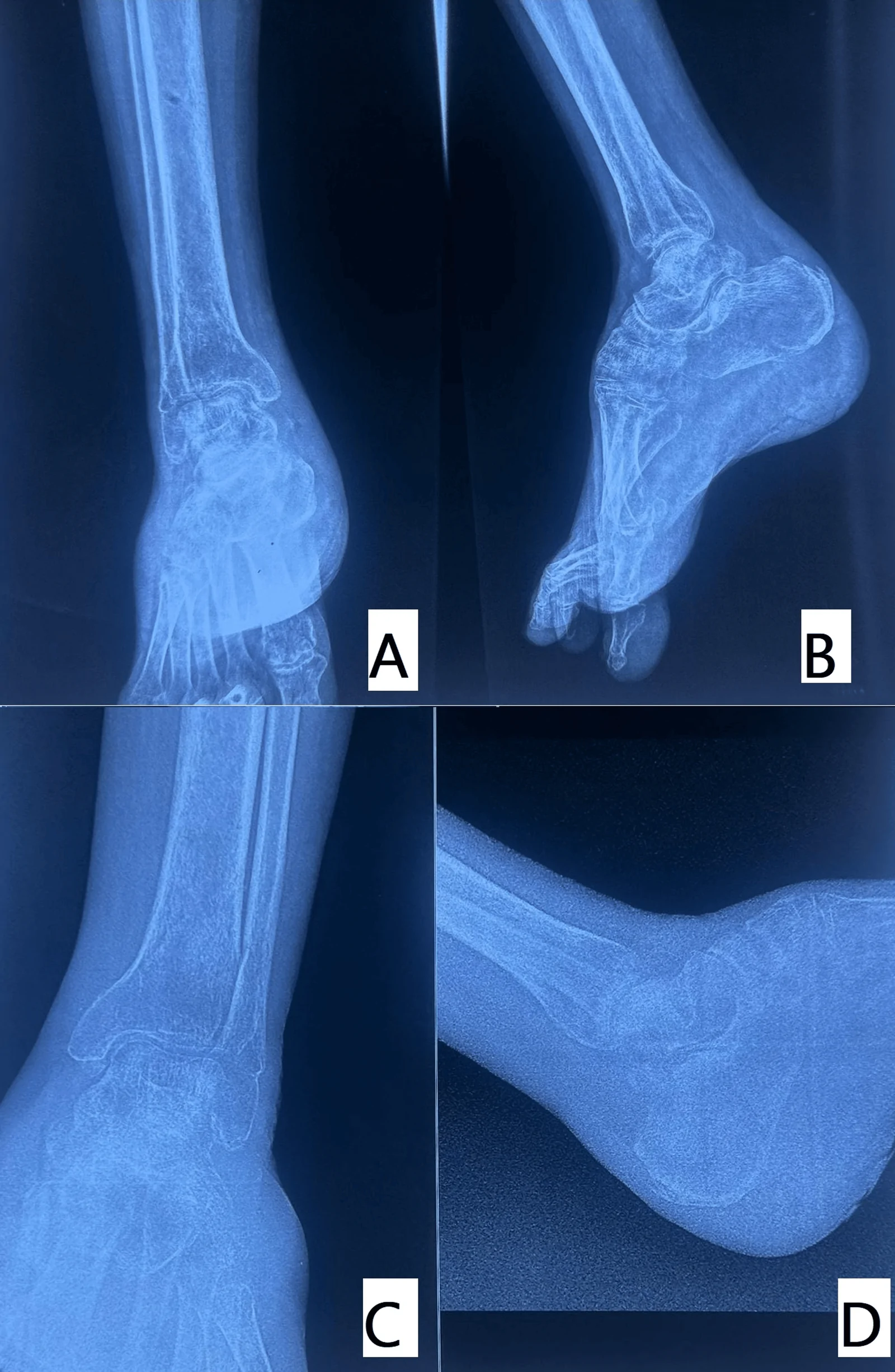
Radiographs at 10 weeks postoperatively after removal of all K-wires (A) Anteroposterior (AP) view of the ankle. (B) Lateral view of the ankle. (C) AP view of the foot and ankle. (D) Lateral view of the foot and ankle demonstrating radiological union.

Following complete hardware removal, the patient was transitioned to a controlled ankle motion (CAM) boot and commenced a structured physical therapy protocol focusing on progressive weight bearing and range-of-motion exercises for the ankle, subtalar, and midfoot joints (Figure 8).

**Figure 8:**
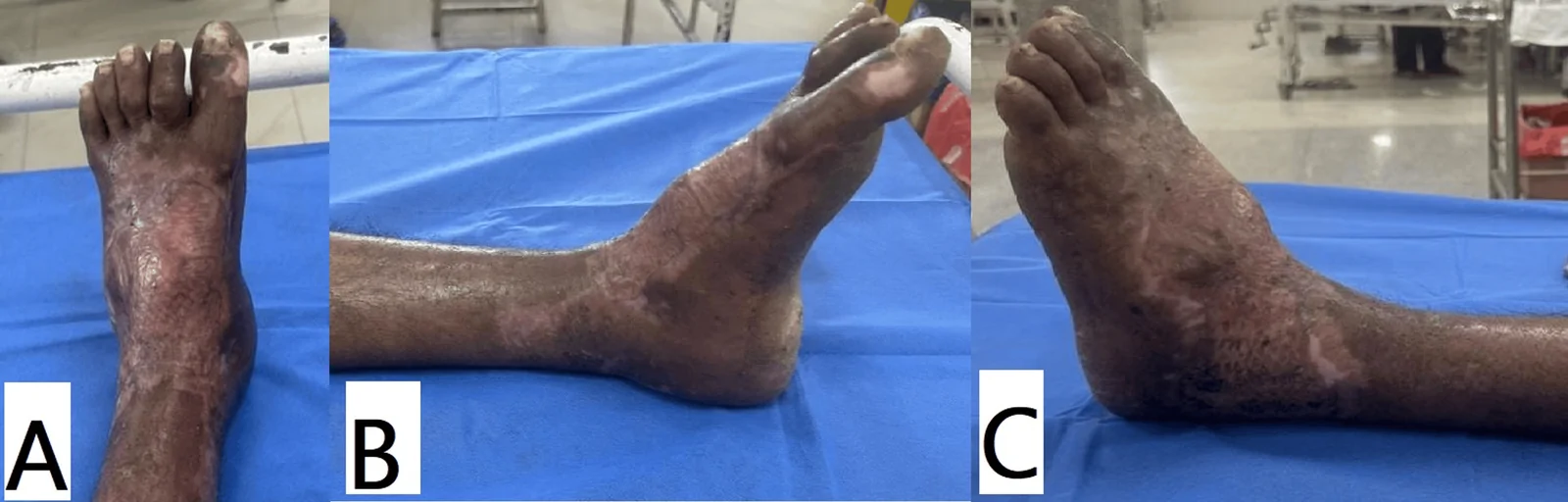
Clinical photographs at follow-up after complete hardware removal and during ongoing rehabilitation (A) Dorsal aspect of the foot. (B) Medial aspect of the ankle and foot. (C) Lateral aspect of the ankle and foot demonstrating final soft-tissue healing and structural alignment of the left lower extremity.

At three months of follow-up, the patient reported minimal pain and was able to ambulate with walker assistance. The soft-tissue envelope was well-healed (Figure 9).

**Figure 9:**
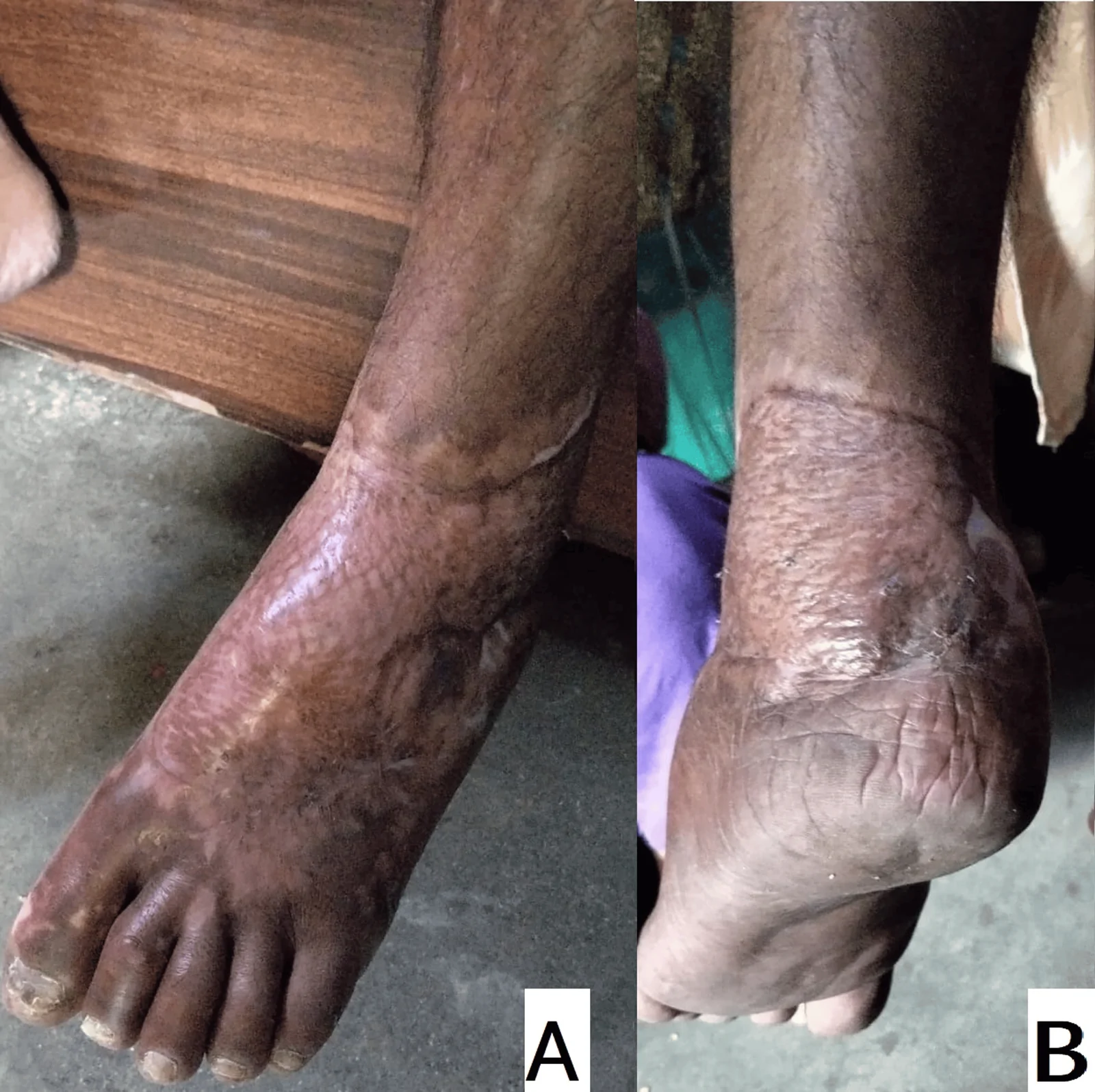
Clinical photographs at three months of follow-up (A) Dorsal aspect of the foot and ankle. (B) Posterior aspect of the ankle demonstrating recovery, maturation of the soft tissues, and structural stability of the left foot and ankle.

Video 1 demonstrates the patient during functional rehabilitation and mobility training three months after surgery.

**Video 1:** Clinical documentation during functional rehabilitation The patient demonstrated safe and stable ambulation using a walker. He achieved a stable, plantigrade stance on the left lower extremity and was able to independently lift, advance, and place the reconstructed foot, indicating satisfactory joint stability. The left foot and ankle demonstrated a mature, well-healed split-thickness skin graft (STSG) over the dorsum and lateral aspect, without evidence of ulceration, wound breakdown, or active infection during ambulation. Facial features were anonymized to preserve patient privacy.

At six months of follow-up, the patient had an AOFAS score of 78 and achieved a functional, plantigrade limb. He was able to perform activities of daily living and return to vocational activities.

## Discussion

The concurrent presentation of a bimalleolar fracture, calcaneal fracture, and Lisfranc injury represents a “floating hindfoot/midfoot” scenario. To our knowledge, few reports have described this exact triad of injuries in combination with a severe open soft-tissue defect.

The primary goal in managing massive open foot and ankle trauma is limb salvage, which depends largely on preservation and survival of the soft-tissue envelope. The traditional gold standard for displaced intra-articular fractures of the calcaneus and bimalleolar ankle, as well as Lisfranc injuries, is anatomic ORIF. However, the use of plates and screws in the presence of a Gustilo-Anderson type IIIB open fracture carries a high risk of deep hardware infection, wound breakdown, and subsequent osteomyelitis.

In this case, the use of K-wires for the midfoot and hindfoot provided low-profile fixation with a minimal footprint. This approach stabilized the osseous architecture without requiring additional periosteal stripping and has been shown to be effective in managing compromised soft-tissue envelopes in calcaneal fractures [1,2]. For the midfoot, although screw fixation may provide greater biomechanical stiffness, K-wires can provide adequate temporary stabilization for Lisfranc injuries without the extensive dissection required for rigid internal fixation [3]. The application of a spanning external fixator was critical; it not only restored length and alignment of the bimalleolar fracture but also provided a rigid scaffold to minimize micromotion of the underlying K-wire construct [4]. By accepting closed or percutaneous reduction and relying on temporary implants as definitive stabilization until union, we avoided the high risk of complications associated with internal fixation in compromised soft tissues. This stepwise approach is consistent with the principles of damage-control orthopedics (DCO), prioritizing physiologic stabilization and soft-tissue preservation over rapid definitive osteosynthesis [5]. The stepwise removal of all hardware further reduced the long-term risks associated with retained foreign bodies beneath a grafted soft-tissue envelope. The patient ultimately achieved a functional, plantigrade limb and was able to perform activities of daily living and return to vocational activities.

## Conclusions

Combined open fractures of the bimalleolar ankle, calcaneus, and Lisfranc joints are extraordinarily rare and limb-threatening. Staged management, prioritizing aggressive initial debridement, percutaneous K-wire fixation, and application of a spanning external fixator, proved to be an effective strategy in this case. This approach protects the fragile vascularity of the foot, permits necessary soft-tissue reconstruction, including STSG, and resulted in a good functional outcome without the morbidity associated with retained internal hardware.
